# Characteristics of *Staphylococcus aureus* Infections, Chicago Pediatric Hospital

**DOI:** 10.3201/eid1302.060295

**Published:** 2007-02

**Authors:** Preeti Jaggi, Suzanne M. Paule, Lance R. Peterson, Tina Q. Tan

**Affiliations:** * Northwestern University, Chicago, Illinois, USA; † Evanston Northwestern Healthcare, Evanston, Illinois, USA

**Keywords:** pediatrics, methicillin-resistant Staphylococcus aureus, Panton-Valentine leukocodin, MRSA, MRSA, dispatch

## Abstract

Invasive and skin community-associated (CA)–methicillin-resistant *Staphylococcus aureus* (MRSA) isolates from children were matched with invasive CA–methicillin-sensitive *S. aureus* strains during 2000–2004. Isolates were analyzed for presence of Panton-Valentine leukocidin. A USA400 lineage clone (n = 6) and the predominant USA300 lineage clone emerged.

Community-associated methicillin-resistant *Staphylococcus aureus* (CA-MRSA) infections have been increasing in children since the 1990s. Panton-Valentine leukocidin (PVL) has been associated with CA-MRSA strains (*1**–**4*).

As CA-MRSA infections have been increasing in previously healthy pediatric patients, we sought to do the following: 1) describe the clonal relatedness of these CA-MRSA isolates by using pulsed-field gel electrophoresis (PFGE), 2) detect the presence of PVL genes among CA-MRSA pediatric isolates causing invasive disease and among isolates causing skin and soft tissue infections (SSTI), 3) determine clinical and epidemiologic differences among patients with invasive disease caused by community-associated methicillin-sensitive *S. aureus* (CA-MSSA) versus those with disease caused by CA-MRSA strains, 4) assess the geographic pattern of infection, and 5) measure the antimicrobial agent susceptibility for CA-MRSA strains.

The institutional review board at Children’s Memorial Hospital, a 253-bed, freestanding children’s hospital in Chicago, Illinois, approved this study. A CA-MRSA strain was defined as a clinical MRSA isolate recovered from a pediatric patient (>1–18 years of age) who had no established risk factors for MRSA infection (no residence in long-term care facility, no hospitalization except for routine birth, and no permanent indwelling medical devices). For most patients, strains were recovered within 72 hours of admission. Exceptions included patients who had clinical evidence of community-associated disease and whose isolates were obtained after 72 hours of hospitalization. Isolates recovered from normally sterile sites were defined as invasive. We identified patients with *S. aureus* infections retrospectively by reviewing microbiology log books from March 1, 2000, through November 30, 2004.

Demographic and clinical data retrieved included age, sex, race, ZIP code of residence, length of hospitalization, and clinical outcomes. When possible, patients with invasive cases were matched to patients with CA-MRSA SSTI and to those with invasive CA-MSSA infections by age (within 12 months for those <18 months or within 3 years for those >18 months), geographic location of patient residence, or year of infection.

*S. aureus* isolates were identified by standard microbiologic methods. For all *S. aureus* isolates that appeared erythromycin resistant and clindamycin susceptible, antibiotic double disk diffusion assay was performed (*5*).

Isolates of *S. aureus,* including control strain NCTC 8325 (Bio-Rad, Hercules, CA, USA), were analyzed by PFGE after DNA digestion with *Sma*I. Resulting fragments were separated by using the *Staphylococcus* program 5 (GenePath System, Bio-Rad), and DNA banding patterns were compared (*6**,**7*). PCR was used to detect the PVL genes (*8*).

Categorical variables were analyzed by χ^2^ analysis. Variables with non-normal distribution were analyzed by Mann-Whitney U test; 2-tailed p value of p<0.05 was statistically significant (SPSS Inc., version 11.0, Chicago, IL, USA).

From 166 MRSA patient strains noted in the microbiology records, 21 patients with invasive CA-MRSA infection were identified. Three patient isolates were unavailable and were excluded from analysis. Patients with invasive CA-MRSA strains were case-matched with patients with CA-MRSA SSTI (16/18 were matched by age). During the study period, ≈31 cases of invasive CA-MSSA disease were identified and 10 cases were able to be retrieved and matched with cases of invasive CA-MRSA (9/10 case-patients were matched by age).

Groups with invasive CA-MRSA and groups with invasive CA-MSSA did not differ significantly regarding sex, initial leukocyte count, duration of fever, or length of hospital stay. Pediatric patients with invasive CA-MRSA infection were more likely to be African American (p = 0.01) and were febrile significantly longer than patients with invasive CA-MSSA (p = 0.03). One of the patients with invasive CA-MRSA died (Table 1).

**Table 1:** Comparison of clinical characteristics between pediatric patients with invasive CA-MRSA and CA-MSSA*

Characteristic	Invasive CA-MRSA, n (%)	Invasive CA-MSSA, n (%)	p value
Race			
Black	16/18 (88)	3/10 (30)	.01
Caucasian	0	2/10 (20)	.01
Hispanic	1/18 (6)	5/10 (50)	.01
Other	0	0	.01
Unknown	1/18 (6)	1/10 (10)	.01
Days of discordant therapy (CA-MRSA)			
Mean ± SD (range)	2.22 ± 1.76 (0–6)	N/A	
Days febrile, mean ± SD (range)	7.0 ± 4 (0–17)	5.20 ± 10.0 (0–32)	.03
Days in hospital, mean ± SD (range)	14.2 ± 7.6 (4–28)	13.8 ± 16.8 (4–60)	NS
Diagnosis†			
Osteomyelitis, acute	3/18 (38)	1/10 (10)	NS
Ostyeomyelitis, chronic	2/18 (11)	4/10 (40)	NS
Bacteremia	5/18 (28)	2/10 (20)	NS
Endocarditis	0/18 (0)	2/10 (20)	NS
Pyomyositis	2/18 (11)	0/10 (0)	NS
Liver abscess	1/18 (6)	0/10 (0)	NS
Pneumonia + empyema	6/18 (33)	1/10 (10)	NS
Septic joint	1/18 (6)	2/10 (20)	NS
Fasciitis	1/18 (6)	0/10 (0)	NS
Toxic-shock syndrome	1/18 (6)	0/10 (0)	NS
Other‡	3/18 (38)	0/10 (0)	NS
Patients with >1 disease manifestation (%)	7/18 (39)	1/10 (10)	NS
Days of illness before hospitalization, mean ± SD (range)	6.9 ± 11.8 (1–45)	10.9 d ± 15 (2–45)	NS
Days of positive cultures, mean ± SD (range)	6.94 ± 9.9 (1–45)	4.4 ± 2.98 (1–11)	NS
Initial leukocyte count‡ (thousand/μL), mean ± SD (range)	14.8 ± 13.07 (2.4–57.0)	9.95 ± 4.45 (3.3–17.0)	NS

*CA-MRSA, community-associated methicillin-resistant *Staphylococcus aureus*; MSSA, methicillin-sensitive *S. aureus;* NS, not significant; SD, standard deviation. Among patients with invasive CA-MRSA infection, 1 died. †Bonferroni correction for multiple comparisons was applied for comparisons of diagnosis. ‡Other diseases included extensive perineal abscess with fistula, subgaleal hematoma, and a subscapular abscess and prostatitis in the same patient. §One initial leukocyte count was unavailable in each patient group.

Among the 18 patients with invasive CA-MRSA, 17 patients required surgical drainage, and 1 patient was given extracorporeal membranous oxygenation. Among the 10 patients with invasive CA-MSSA disease, all required surgical intervention, but none died. Of 18 patients with SSTI CA-MRSA disease, 12 were African American, and 2 were Hispanic. The average hospital stay for this group was 2.3 days. Seven children required no hospitalization or hospitalization <24 hours.

A representative PFGE result is depicted in the Figure. Two predominant clones emerged among the local and invasive CA-MRSA isolates. The clone A (n = 6) was identified to be of the USA400 lineage, and the “B” clone (n = 30) was identified to be of the USA300 lineage. No predominant clones emerged from the invasive MSSA isolates; 6 unique clones were identified from 10 isolates (data not shown). No isolates were of the USA300 or 400 lineage. The clinical manifestations of invasive disease in patients with CA-MRSA disease from clone A were pneumonia with empyema, osteomyelitis, bacteremia, and septic arthritis. The clinical manifestations of invasive disease in patients with clone B infection included sepsis, toxic-shock syndrome, osteomyelitis, fasciitis, bacteremia, pyomyositis, pneumonia with empyema, deep visceral abscesses, and perirectal abscess with prostatitis; 1 patient in this group died. Clone A was found in a wide geographic distribution around Chicago. In contrast, clone B was located with more frequency within the city limits of Chicago.

**Figure:**
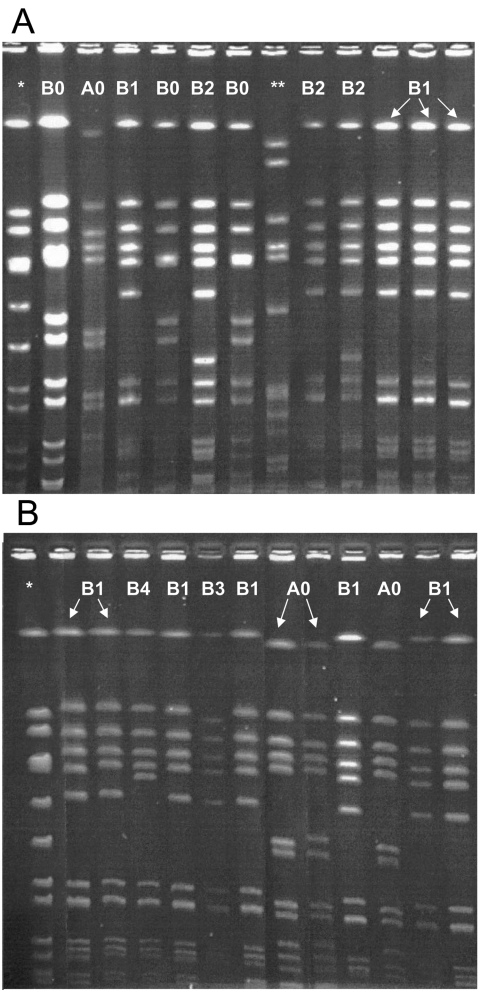
Pulsed-field gel electrophoresis (PFGE) results for community-associated methicillin-resistant *Staphylococcus aureus* (MRSA) isolates causing disease. A) Local skin and soft tissue isolates. B) Invasive isolates. *, PFGE control; **, clinical isolate, hospital-associated MRSA.

Antimicrobial susceptibility patterns among CA-MRSA strains are detailed in Table 2. Two CA-MRSA isolates that were positive by 3-D test belonged to the A0 clone. None of the MRSA isolates that caused SSTI was D-test positive. The PVL gene was found in all of the CA-MRSA isolates that caused invasive and SSTI disease but in only 1 of 10 of the invasive CA-MSSA isolates (p<0.001).

**Table 2:** Susceptibility data for CA-MRSA isolates from children*

Susceptibility to antimicrobial agent†	CA-MRSA isolates causing invasive disease, n (%)	CA-MRSA isolates causing local skin/soft tissue infections, n (%)	p value
Resistant to erythromycin	16/18 (88.9)	11/18 (61)	NS
Apparently susceptible to clindamycin	18/18 (100)	18/18 (100)	NS
Inducible clindamycin resistance	3/16 (19)	0/10 (0)	NS
Resistant to ciprofloxacin	2/18 (11)	1/18 (6)	NS
Resistant to levofloxacin	1/18 (6)	1/18 (6)	NS
Resistant to tetracycline	1/18 (5)	2/18 (11)	NS

*CA-MRSA, community-associated methicillin-resistant *Staphylococcus aureus*; NS, not significant. †In addition to the antimicrobial agents listed, all isolates were susceptible to vancomycin, linezolid, and rifampin.

## Discussion

PVL genes can be transmitted by means of bacteriophages, which allows them to be transmitted from 1 organism to another (*9*). When injected intradermally in rabbits, PVL induces necrotic skin lesions (*10*), and PVL has been described in *S. aureus* isolates from patients with necrotizing pneumonia, skin infections, and musculoskeletal infections. These outbreaks have been widespread (*1**,**2**,**8**,**11**–**13*). We found a wide range of severity of infection caused by clonally related CA-MRSA, PVL-positive isolates within our community, from superficial skin abscesses to fatal disease.

Results of PFGE correlate well with results of other molecular typing methods, such as multilocus sequence typing (MLST), which characterizes *S. aureus* species by using sequences of 7 conserved housekeeping genes (*7*). In accordance with other published studies in the United States, the USA300 strain was the most frequently isolated among CA-MRSA disease (*12*) in contrast to the invasive CA-MSSA disease, which had no predominant clonality. We also detected isolates of the USA400 lineage that harbored PVL genes. This lineage was previously described as a cause of severe and fatal CA-MRSA disease in children in the Midwest (*14*). Previous reports from University of Chicago have described a cluster of 4 cases in which USA400 isolates caused empyema and sepsis syndrome with some features of toxic-shock syndrome (*4*); 3 children died with necrotizing pneumonia and Waterhouse-Friderichsen syndrome due to a PVL-positive, MLST-identified type 1 strain (*15*). In contrast, our patient with fatal toxic-shock syndrome did not have any primary pulmonary pathology and had disease caused by a USA300 lineage strain.

Limitations to our study include its retrospective nature and the limited numbers of patients. The geographic distribution of CA-MRSA isolates within the city likely reflects the geographic distribution of our patient population. Future prospective studies may further elucidate possible epidemiologic risk factors associated with acquiring CA-MRSA invasive infection.

## References

[1] Baggett HC, Hennessy TW, Rudolph K, Bruden D, Reasonover A, Parkinson A, Community-onset methicillin-resistant Staphylococcus aureus associated with antibiotic use and the cytotoxin Panton-Valentine leukocidin during a furunculosis outbreak in rural Alaska. J Infect Dis. 2004;189:1565–73. 10.1086/383247 15116291

[2] Martinez-Aguilar G, Avalos-Mishaan A, Hulten K, Hammerman W, Mason EO Jr, Kaplan SL, Community-acquired, methicillin-resistant and methicillin-susceptible Staphylococcus aureus musculoskeletal infections in children. Pediatr Infect Dis J. 2004;23:701–6. 10.1097/01.inf.0000133044.79130.2a 15295218

[3] Mishaan AM, Mason EO Jr, Martinez-Aguilar G, Hammerman W, Propst JJ, Lupski JR, Emergence of a predominant clone of community-acquired Staphylococcus aureus among children in Houston, Texas. Pediatr Infect Dis J. 2005;24:201–6. 10.1097/01.inf.0000151107.29132.70 15750454

[4] Mongkolrattanothai K, Mason EO Jr, Martinez-Aguilar G, Hammerman W, Propst JJ, Lupski JR, Severe Staphylococcus aureus infections caused by clonally related community-acquired methicillin-susceptible and methicillin-resistant isolates. Clin Infect Dis. 2003;37:1050–8. 10.1086/378277 14523769

[5] Fiebelkorn KR, Crawford SA, McElmeel ML, Jorgensen JH. Practical disk diffusion method for detection of inducible clindamycin resistance in Staphylococcus aureus and coagulase-negative staphylococci. J Clin Microbiol. 2003;41:4740–4. 10.1128/JCM.41.10.4740-4744.2003 14532213 PMC254362

[6] Tenover FC, Arbeit RD, Goering RV, Mickelsen PA, Murray BE, Persing DH, Interpreting chromosomal DNA restriction patterns produced by pulsed-field gel electrophoresis: criteria for bacterial strain typing. J Clin Microbiol. 1995;33:2233–9. 7494007 10.1128/jcm.33.9.2233-2239.1995 PMC228385

[7] McDougal LK, Steward CD, Killgore GE, Chaitram JM, McAllister SK, Tenover FC. Pulsed-field gel electrophoresis typing of oxacillin-resistant Staphylococcus aureus isolates from the United States: establishing a national database. J Clin Microbiol. 2003;41:5113–20. 10.1128/JCM.41.11.5113-5120.2003 14605147 PMC262524

[8] Lina G, Piemont Y, Godail-Gamot F, Bes M, Peter MO, Gauduchon V, Involvement of Panton-Valentine leukocidin-producing Staphylococcus aureus in primary skin infections and pneumonia. Clin Infect Dis. 1999;29:1128–32. 10.1086/313461 10524952

[9] Narita S, Kaneko J, Chiba J, Piemont Y, Jarraud S, Etienne J, Phage conversion of Panton-Valentine leukocidin in Staphylococcus aureus: molecular analysis of a PVL-converting phage, phiSLT. Gene. 2001;268:195–206. 10.1016/S0378-1119(01)00390-0 11368915

[10] Ward PD, Turner WH. Identification of staphylococcal Panton-Valentine leukocidin as a potent dermonecrotic toxin. Infect Immun. 1980;28:393–7. 7399669 10.1128/iai.28.2.393-397.1980 PMC550947

[11] Prevost G, Couppie P, Prevost P, Gayet S, Petiau P, Cribier B, Epidemiological data on Staphylococcus aureus strains producing synergohymenotropic toxins. J Med Microbiol. 1995;42:237–45. 7707330 10.1099/00222615-42-4-237

[12] Francis JS, Doherty MC, Lopatin U, Johnston CP, Sinha G, Ross T, Severe community-onset pneumonia in healthy adults caused by methicillin-resistant Staphylococcus aureus carrying the Panton-Valentine leukocidin genes. Clin Infect Dis. 2005;40:100–7. 10.1086/427148 15614698

[13] Kazakova SV, Hageman JC, Matava M, Srinivasan A, Phelan L, Garfinkel B, A clone of methicillin-resistant Staphylococcus aureus among professional football players. N Engl J Med. 2005;352:468–75. 10.1056/NEJMoa042859 15689585

[14] Centers for Disease Control and Prevention. Four pediatric deaths from community-acquired methicillin-resistant Staphylococcus aureus—Minnesota and North Dakota, 1997–1999. MMWR Morb Mortal Wkly Rep. 1999;48:707–10. 21033181

[15] Adem PV, Montgomery CP, Husain AN, Koogler TK, Arangelovich V, Humilier M, Staphylococcus aureus sepsis and the Waterhouse-Friderichsen syndrome in children. N Engl J Med. 2005;353:1245–51. 10.1056/NEJMoa044194 16177250

